# Longitudinal study of gastroesophageal reflux and erosive tooth wear

**DOI:** 10.1186/s12876-017-0670-1

**Published:** 2017-10-25

**Authors:** Clive H. Wilder-Smith, Andrea Materna, Lukas Martig, Adrian Lussi

**Affiliations:** 1 Brain-Gut Research Group and Gastroenterology Group Practice, Bubenbergplatz 11, -3011 Bern, CH Switzerland; 2 Department of Preventive, Restorative and Pediatric Dentistry, University of Bern, Bern, Switzerland; 3 Institute of Mathematical Statistics and Actuarial Science, University of Bern, Bern, Switzerland

**Keywords:** Dental erosion, Gastroesophageal reflux, GERD, Proton pump inhibitor, Esomeprazole, pH-impedance

## Abstract

**Background:**

Approximately 60% of patients presenting to dentists with erosive tooth wear have significant gastroesophageal reflux (GERD), despite minor reflux symptoms. No longitudinal studies of reflux-associated erosive tooth wear and of reflux characteristics have been reported to date.

The aim of this study was to characterize the longitudinal course of GERD and of associated erosive tooth wear, as well as factors predictive of its progression, in a large group of patients.

**Methods:**

Seventy-two patients presenting to dentists with clinically significant erosive tooth wear and increased esophageal acid exposure by 24-h multichannel intraluminal pH-impedance measurement (MII-pH) were re-assessed clinically and by MII-pH after 1 year treatment with esomeprazole 20 mg twice-daily. Predictive factors for erosive tooth wear were assessed by logistic regression.

**Results:**

At follow-up, no further progression in erosive tooth wear was observed in 53 (74%) of patients. The percentage of time with a pH < 4, the number of acid reflux episodes and the percentage of proximal esophageal reflux off-PPI did not change significantly after one year, but the number of weakly acidic reflux episodes decreased significantly in the large subgroup without progression. None of the baseline demographic, clinical, endoscopic or esophageal acid exposure characteristics were significantly associated with progression of erosive tooth wear at follow-up.

**Conclusions:**

In this longitudinal study in patients with erosive tooth wear and oligosymptomatic GERD receiving esomeprazole for one year, erosive tooth wear did not progress further in the majority of patients. Background acidic esophageal reflux exposure appeared stable over time, whereas weakly acidic exposure decreased significantly in patients without erosion progression. MII-pH measurements on-PPI and with healthy controls will be useful in the further elucidation of the causal role of reflux in erosive tooth wear.

**Trial registration:**

ClinicalTrials.gov, retrospectively registered: NCT02087345.

## Background

Dental erosion, the chemical dissolution of enamel without bacterial involvement, is considered an established complication of gastroesophageal reflux (GERD) [1]. It is caused by repeated episodes of exposure to acid and is influenced by chemical (e.g. salivary buffering effects, pH, fluoride,) and physical (temperature, flow rate) factors [2]. As dental erosion commonly occurs in conjunction with abrasive processes, it is now termed “erosive tooth wear” [3]. Advanced erosive tooth wear leads to functional compromise, oral symptoms and disfigurement. The reported prevalence erosive tooth wear varies between 17% and 68% in patients with symptomatic GERD and a recent study of erosive tooth wear revealed a prevalence of 29% in 3187 European adults aged 18–35 years without symptomatic GERD [4, 5]. Recently, characterisation of reflux by 24-h multichannel intraluminal pH-impedance measurements (MII-pH) and endoscopy in a large prospective cohort of oligosymptomatic patients presenting with erosive tooth wear to dentists was published, showing significant gastroesophageal reflux in the majority [5]. This further substantiated the frequent association between GERD and erosive tooth wear, after exclusion of other causes of erosion, such as frequent vomiting, increased ingestion of acidic food or drink and bruxism. Once erosive tooth wear is established, prevention of further loss of dental tissue is important, not only because of the high cost of the necessary dental work, but also because of the common sequelae of oral hypersensitivity, functional and aesthetic impairment. Once clinically manifest, erosive tooth wear is currently not reversible. To the best of our knowledge, no systematic study relating to the prevention of progression of erosive tooth wear caused by GERD has been reported, except a small pilot project examining optical coherence tomography as a tool for quantifying dental reflux damage [6]. As no reliable local preventive treatment exists, our centre has in the past arbitrarily recommended long-term proton pump inhibitor (PPI) dosing. In this article we present single-centre, follow-up data regarding GERD characteristics using MII-pH and erosive tooth wear progression in a large cohort of patients receiving standardised PPI treatment, based on the hypothesis that reflux would not significantly change over time and that standard PPI doses would prevent further progression of erosions. Predictive factors for the progression of erosions were additionally investigated.

## Methods

**Fig. 1:**
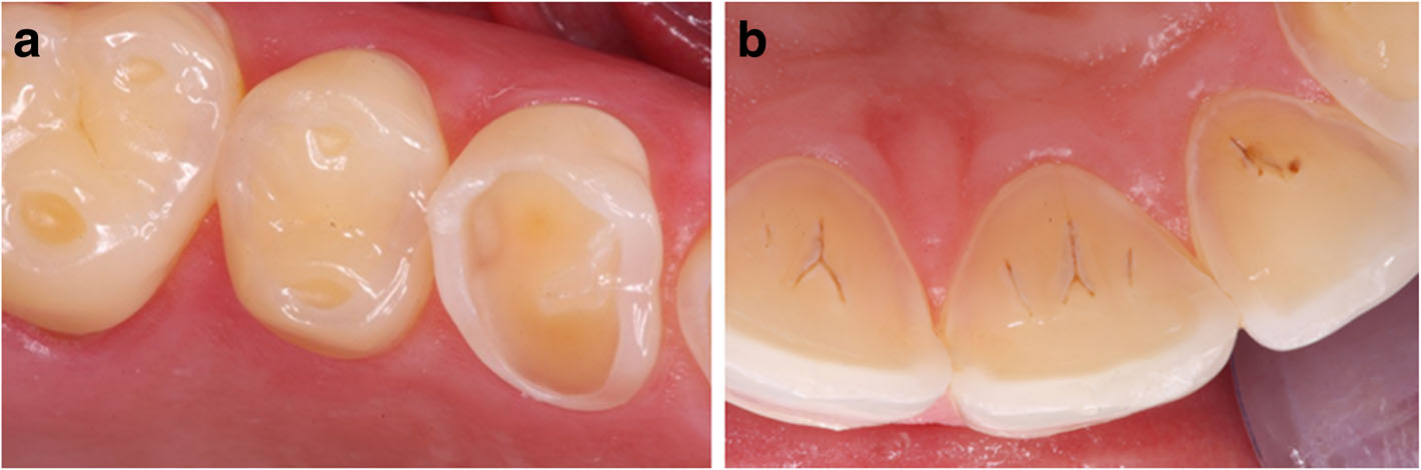
Typical high-resolution photographs of dental erosions secondary to gastroesophageal reflux. **a** Oral (BEWE 3). **b** Occlusal (BEWE 3). BEWE = basal erosive wear examination [7]

Consecutive patients presenting at the Department of Preventive, Restorative and Pediatric Dentistry of the University of Bern and affiliated dentists with an erosive tooth wear score greater than 8 (BEWE: Basic Erosive Tooth Wear Examination) – see below for definition), were referred to the Gastroenterology Group Practice between 1/2010 and 12/2012 for evaluation of GERD after exclusion of non-reflux causes of erosion by detailed medical history, dental examination, a standardized dietary diary, and measurement of salivary flow and buffering capacity using standard procedures [7, 8]. Patients with a history of bruxism, eating disorders, recurrent vomiting, severe obesity (body mass index (BMI) > 35 kg/m^2^) or past bariatric surgery (both because of the likelihood of representing a specialized subgroup of GERD), and dietary or primarily abrasive causes for erosive tooth wear were excluded. All patients were seen by a dedicated team of dentists experienced in the diagnosis of erosion and the severity of erosion was graded using the BEWE [7]. The BEWE is a simple cumulative scoring system quantifying the size of a given lesion as a percentage of the surface affected. The vestibular, occlusal and palatal surfaces of all teeth except third molars are graded. The dentition is divided into sextants, the most severe score in a sextant is recorded and the cumulative score from all sextants (maximum = 18) represents the index value. Further, high-quality photographs of the teeth were examined and the findings recorded (Fig. 1). Upon referral, every patient was examined by the same senior gastroenterologist (CWS), reflux symptoms were assessed by interview and the Reflux Disease Questionnaire and esophago-gastro-duodenoscopy with gastric and, if clinically indicated, esophageal biopsies, and subsequent 24-h oesophageal MII-pH (Ohmega, MMS, Enschede, Holland) were performed [9].The single-use MII-pH catheter (pHersaflex, Sierra Scientific Instruments, Los Angeles, USA) with impedance measurement sites at 3, 5, 7, 9, 15 and 17 cm and a distal pH-sensor at 5 cm above the lower oesophageal sphincter was introduced transnasally after an overnight fast and placed with the pH sensor at 5 cm proximal to the oesophago-gastric junction. Patients were instructed to perform their normal daily activities, eat and drink as usual, and document the times at which they lay down, ate and drank or experienced reflux symptoms using the datalogger’s event markers. Patients on antisecretory medication discontinued their dosing 14 days before the MII-pH recording. The numbers of all (pH < 7), acidic (pH < 4) and weakly acidic (pH >4 and <7) reflux episodes, the percentage time with pH < 4 and <5.5, the percentage of proximal reflux episodes (reaching 15 cm above the gastroesophageal junction) in the total 24 h, the DeMeester score and the symptom associations by symptom association probability (SAP) >95% were analyzed [9, 10]. Patients with a pH < 4 for more than 5% of the measurement period or more than 75 total (acidic plus weakly acidic), 50 acidic and 33 weakly acidic reflux episodes at 5 cm and more than 30 total reflux episodes at 15 cm above the gastroesophageal junction were considered to have increased reflux based on normal European values published by Zerbib et al. [11].

Patients with increased reflux at the first work-up received prescriptions for esomeprazole 20 mg tablets (AstraZeneca AG, Zug, Switzerland), to be taken 10–20 min before breakfast and dinner. This dose was chosen as the most effective PPI dosing regimen based on published pharmacodynamic data [12, 13]. These patients were recalled for the same detailed dental assessment as outlined above within 2 years. Compliance with the treatment was assessed by verbal interview and was rated as high (dose missed on average less than once per week), moderate (dose missed between once and three times per week) or poor (dose missed more than three times per week). Patients were also questioned about any adverse events they had experienced during treatment with the PPI.

This was a study analyzing coded clinical data with no additional research-related procedures for which no Cantonal Ethics Committee approval or written informed consent were required in Switzerland at the time, until the institution of the Human Research Law (Humanforschunggesetz) after 1.2014. The study was conducted according to the principles of the 1964 Declaration of Helsinki and its later amendments and patients provided verbal informed consent.

### Statistical analysis

Analyses were performed with the statistical Software R, Version 3.0.1 (http://www.R-project.org, Revolution Analytics, Vienna, Austria) by the biostatistical co-author. The means and 95% confidence intervals of normally distributed variables, and medians and interquartile ranges of non-normally distributed or non-continuous data are shown. Erosion progression at follow-up within the first 2 years was the target value and was defined as an increased BEWE score compared to baseline. Progression was assessed as either ‘yes’ or ‘no’, and therefore was a dichotomous value. We assessed the impact of the following baseline describing values on the target value: gender, age, GERD symptoms less or more than twice a week, the presence of a hiatal hernia, the percentage of reflux episodes reaching the proximal oesophagus, the BEWE score, the percentage of time with pH < 4 and <5.5, the DeMeester score and the numbers of acidic and weakly-acidic reflux episodes. The impact of the dichotomous describing values, namely gender, GERD symptoms and the presence of a hiatal hernia, was assessed using Fisher’s exact test. The impact of the above metric variables was assessed using a logistic regression model. Multiple testing was corrected for using Holm’s methodology. Between group comparisons of MII-pH data were performed with the Mann Whitney U test for non-parametric and the t-test for parametric variables.

## Results

During the recruitment period 161 patients presenting with erosive tooth wear underwent baseline work-up as described in the Methods section. 141 of the 161 patients (88%) included in the baseline work-up had increased esophageal acid exposure compared to published normal values [11]. 72 of the 161 patients (45%) had increased reflux and returned for the scheduled follow-up within 2 years. Of the 89 patients excluded from the study, 19 had a normal MII-pH analysis at baseline and 70 patients with an abnormal MII-pH at baseline were excluded for the following reasons: 49 had a change of dentist or the dentist failed to provide comparative erosion data, 13 patients were lost to follow-up and could not be contacted and 8 patients refused the follow-up examination. There were no statistically significant differences in baseline demographics, GERD characteristics or MII-pH results between those patients who qualified for follow-up but dropped out versus those who completed follow-up. Seventy-two patients with erosive tooth wear and abnormal reflux at baseline were therefore included in this longitudinal analysis. Their characteristics are summarized in Table 1. Fifty-nine of the patients were assessed 1 year after the baseline assessment, one patient after 7 months and 12 patients after 2 years.

**Table 1:** Baseline characteristics of 72 patients presenting with dental erosion and treated with esomeprazole 20 mg twice-daily

Gender: male/female (n)	52 / 20
Age: years#	33.8 (29.1–38.5)
Patients with GERD symptoms >2 per week (n)	15
Reflux Disease Questionnaire score+	3 (1–5)
Patients previously using PPI regularly (n)	0
Patients with endoscopic hiatal hernia (n)	12
BEWE score+	14 (11–17)
Median follow-up time+	1 (1–1)

*BEWE* Basic erosive wear examination [7]. (9–13 medium, >13 extensive erosive disease), *GERD* gastrooesophageal reflux disease, *PPI* proton pump inhibitor. #means (95% confidence intervals) + median (interquartile range)

No progression of erosive tooth wear was observed in 53 of the 72 patients (74%), while progression was seen in 19 (26%) within the follow-up period with esomeprazole treatment. None of the baseline demographic, clinical, endoscopic or 24 h–MII-pH characteristics were significantly associated with progression of erosive tooth wear at follow-up.

The MII-pH data at baseline and at follow-up after a median interval of 1 year are shown in Table 2. The percentage of time with reflux below thresholds of pH 4 and 5.5, and the percentage of proximal esophageal reflux were not significantly different from baseline after 1 year. However, the number of weakly acidic reflux episodes was significantly decreased at follow-up, explaining the reduction in the number of all reflux episodes. These decreases from baseline were significant in the subgroup of patients with no progression of erosion, but did not reach significance in the smaller subgroup of patients with progression of erosions. Analysis of the correlation between symptoms and reflux episodes during MII-pH by SAP, SII and SI was not relevant, as 86% of patient did not report reflux symptoms during MII-pH.

**Table 2:** Twenty-four-hour ambulatory multichannel pH-impedance measurement results and BEWE scores at baseline and at follow-up off-PPI in patients with progression or no progression of dental erosion after a median of 1 year treatment with esomeprazole 20 mg twice-daily. Means and 95% confidence intervals are shown

	At baseline	At follow-up		
	All patients N = 72	All patients *N* = 72	No erosion progression *N* = 53	Erosion progression *N* = 19
% time with pH < 4^a^	16.5 (12.6–20.4)	11.7 (5.7–17.7)	10.2 (6.7–13.7)	16.9 (6.4–27.4)
% time with pH < 5.5^a^	43.5 (33.3–53.7)	40.2 (31.1–49.3)	40.5 (26.6–54.4)	37.7 (26.6–48.8)
DeMeester score^a^	56.4 (43.2–69.6)	46.3 (34.3–54.3)	41.7 (31.8–51.6)	69.1 (40.1–98.1)
Number of all reflux episodes (pH < 7)^b^	85 (64–106)	52 (41–63)*	53 (46–61)**	52 (33–71)
Number of acidic reflux episodes (pH < 4)^b^	54 (41–67)	41 (31–50)	43 (37–49)	33 (24–42)
Number of weakly acidic reflux episodes (pH > 4–pH < 7)^b^	31 (24–38)	12 (8–18)**	10 (8–12)**	19 (10–28)
% proximal reflux (15 cm above lower oesophageal sphincter)^a^	26 (20–32)	23 (17–29)	23 (19–27)	21 (15–27)
BEWE scores^b^	12 (11–16)	13 (12–15)	12 (11–13)	14 (13–16)***

*BEWE* Basic erosive wear examination (9–13 medium, >13 extensive erosive disease) [7]

^a^means (95% confidence intervals)

^b^median (interquartile range)

*p < 0.05 versus baseline **p < 0.01 versus baseline ***p < 0.01 group with erosion progression vs group without erosion progression

No significant adverse events necessitating discontinuation of esomeprazole were noted. Compliance was rated high in 86% of patients, moderate in 12% and poor in 2%.

## Discussion

This longitudinal 1-year study characterized the course of erosive tooth wear and of gastroesophageal reflux by MII-pH in patients initially presenting to dentists. It corroborates earlier data showing the high prevalence of oligosymptomatic reflux (88% in this study) in patients presenting to dentists with erosive tooth wear [5]. Approximately 80% of these patients do not complain of relevant reflux symptoms. The course of established erosive tooth wear over time is relatively unknown, especially in patients with proven GERD.

The current investigation aimed to provide further information in this little-studied group of patients. Erosive tooth wear progressed in 26% of our adult patients with GERD after a median follow-up of one year. Esomeprazole was prescribed to all patients at baseline for prevention of progression of erosive tooth wear, as no treatment guidelines have thus far been formulated. The absence of further progression of erosion during active GERD may be due to several factors. These include the protective anti-secretory action of the PPI, a spontaneous decrease in reflux (natural history), or an association with unrecognized factors other than acid reflux, for example, abnormalities in the saliva. A few prospective studies in healthy adolescents, i.e. without known GERD, describe progression of erosion in between 3.5% and 18% patients annually, and in 27% and 56% after 2 to 4 years using various clinical grading scales and methodologies [14–17]. In a prospective study using silicone impressions, over 70% of subjects had progressive tooth substance wear of over 15 μm (accuracy threshold of measurement, but unknown relationship to visual BEWE scoring) over a 6-month period. This was a mixed cohort of subjects, including some individuals with symptomatic reflux [18]. These cohorts cannot be used as direct controls; however, the erosion progression in healthy controls appears considerably lower than in our group with proven GERD. It is evident, that some progressive loss of tooth substance is normal with ageing and that this loss is more likely to occur in bursts than at a constant rate [18]. One underlying cause of such bursts may well be GERD.

There is also little data on the natural history of silent or primarily supra-esophageal reflux, although using clinical and endoscopic assessments, symptomatic GERD appears to be a chronic and stable disease, with regression in the severity of manifestations occurring in a minority of patients [19–22]. This observation is, however, of limited applicability to oligosymptomatic patients, and relevant longitudinal MII-pH data is lacking. In the current study acidic reflux characterized by MII-pH was stable and did not differ between patients with and without erosive tooth wear progression. However, weakly acidic acid exposure fluctuated considerably over time, showing a significant decrease at follow-up in the entire group of patients and in the large subgroup without erosion progression. There was also a corresponding clear trend in patients with erosion progression, but due to the smaller sample size, significance compared to baseline was not attained. Consequently, esophageal exposure to weakly acidic refluxate fluctuates more than to acidic refluxate with a pH below 4. Nonetheless, this background fluctuation in the natural history of GERD is unlikely to explain the absence of erosion progression in a subgroup of patients. It should be noted that the repeatability of MII-pH has been examined and is improbable as an explanation of the variation in acid exposure [11].

None of the baseline demographic, endoscopic or acid exposure characteristics predicted subsequent progression of erosive tooth wear. This implies that baseline characteristics do not predict the response of erosive tooth wear to the PPI treatment, but this could only be confirmed by the inclusion of a control group without treatment, which is very difficult for ethical reasons. Interestingly, clinical variables and acid exposure characteristics by MII-pH reflux variables also do not significantly predict the symptomatic response to PPI’s in GERD, as shown by Zerbib et al. [23]. This suggests that factors other than refluxed acid alone, such as proteases and bile in refluxate or non-reflux associated factors, may play a prominent role in erosive tooth wear. A MII-pH study during PPI treatment would provide further relevant information on the longitudinal relationship between erosion progression and reflux.

The major limitation of this study is the absence of an untreated control group, which had in a related, earlier study been considered unethical by the responsible review board. An inherent methodological limitation in all in vivo dental studies is the detailed quantification of erosive tooth wear. However, the best possible standardization of dental evaluations was achieved by having the same dentist from a highly specialized university department assess the same patient at baseline and follow-up, using the recently defined BEWE scoring system as well as high-resolution photography. The BEWE is a scoring system with three severity grades. Minor increases in erosion are less likely to be reflected in the scores, or, indeed, to be detected visually. Information on the smoking or alcohol consumption, further confounders in GERD studies, was not available in this study.

## Conclusions

In summary, after a median follow-up of one year, erosive tooth wear did not progress further in 74% of patients with erosive tooth wear and oligosymptomatic GERD receiving esomeprazole. Baseline demographic, clinical and MII-pH date were not predictive of progression of erosive tooth wear. Background acidic esophageal reflux exposure appeared stable over time, whereas weakly acidic exposure decreased significantly in patients without erosion progression. MII-pH measurements on-PPI and with healthy controls are required to further examine the causal role of reflux in dental erosion.

## Abbreviations

BEWE: Basic erosive wear examination
GERD: Gastroesophageal reflux
MII-pH: 24-h ambulatory multichannel pH-impedance measurement
PPI: Proton pump inhibitor
SAP: Symptom association probability
